# Strengthening TB laboratory systems: addressing diagnostic and systemic barriers in drug-resistant TB

**DOI:** 10.5588/pha.25.0009

**Published:** 2025-06-04

**Authors:** J.-K. Jung, M. Quelapio, S.N. Cho

**Affiliations:** ^1^ International Tuberculosis Research Center, 236 Gaposunhwan-ro, Masanhappo-gu, Changwon-si, Gyeongsangnam-do, Republic of Korea;; ^2^ KNCV TB Foundation, Postbus 146, 2501 CC Den Haag, The Hague, The Netherlands;; ^3^ TB Alliance, 40 Wall Street, 24th Floor, New York, USA.

**Keywords:** tuberculosis, TB laboratory network, diagnostic challenges, health system approach, LIFT-TB

## Abstract

As part of the LIFT-TB project, we investigated the efficacy and safety of the BPaL regimen, while enhancing laboratory diagnostic capacity for drug-resistant-TB. Challenges include sample contamination, excessive workload, test kit shortages, unreliable results and systemic issues such as infrastructure, delayed procurement, workforce constraints and reliance on paper-based data reporting. Our study highlights the need for a system-level approach backed by strong national leadership to strengthen TB diagnostic capacity. Although countries must take ownership of laboratory system improvements, a harmonized and coordinated approach among international stakeholders is essential in specialized areas, such as external quality assurance, capacity building, and introducing innovative diagnostic technologies.

The ‘Leveraging Innovation for Faster Treatment of Tuberculosis’ (LIFT-TB) project is an operational research (OR) program launched in late 2020 and successfully implemented in seven countries, including the Philippines, Myanmar, Indonesia, Vietnam, Uzbekistan, Kyrgyzstan, and Ukraine.^1^ The project aimed to investigate the efficacy and safety of the BPaL (bedaquiline, pretomanid, and linezolid) regimen to treat pre-extensively drug-resistant forms of pulmonary TB (pre-XDR-TB) or treatment-intolerant or non-responsive multi-drug resistant TB (MDR-TB) patients. During the project, the International Tuberculosis Research Center (ITRC) took responsibility for enhancing the diagnostic capacity of mycobacterial laboratories participating in the OR (the labs included national, subnational reference laboratories, and peripheral laboratories performing sputum culture and drug susceptibility testing and molecular tests). Here we highlight the laboratory-related challenges and propose more systemic and harmonized approaches to address these.

## LABORATORY-RELATED CHALLENGES

### Testing process issues

In the laboratory cascade from sputum collection to release of test results, multiple challenges were identified in TB laboratories (while also acknowledging variability in the magnitude of these challenges among the participating laboratories). These issues included poor quality of sputum samples (contaminated during collection and testing), delays in transportation and loss of samples, stockouts of essential test kits, and the risk of TB samples being unattended due to excessive workload (see Figure). Moreover, even when tests were conducted, some results could be unreliable due to errors (e.g., reagent handling, instrument maintenance) and further delays in reporting affected timely treatment initiation. This challenge aligns with recent WHO recommendations on TB diagnostics, which emphasize the importance of quality assurance programs, digital data reporting systems, and training for laboratory personnel to enhance diagnostic reliability.^2^

**FIGURE.**
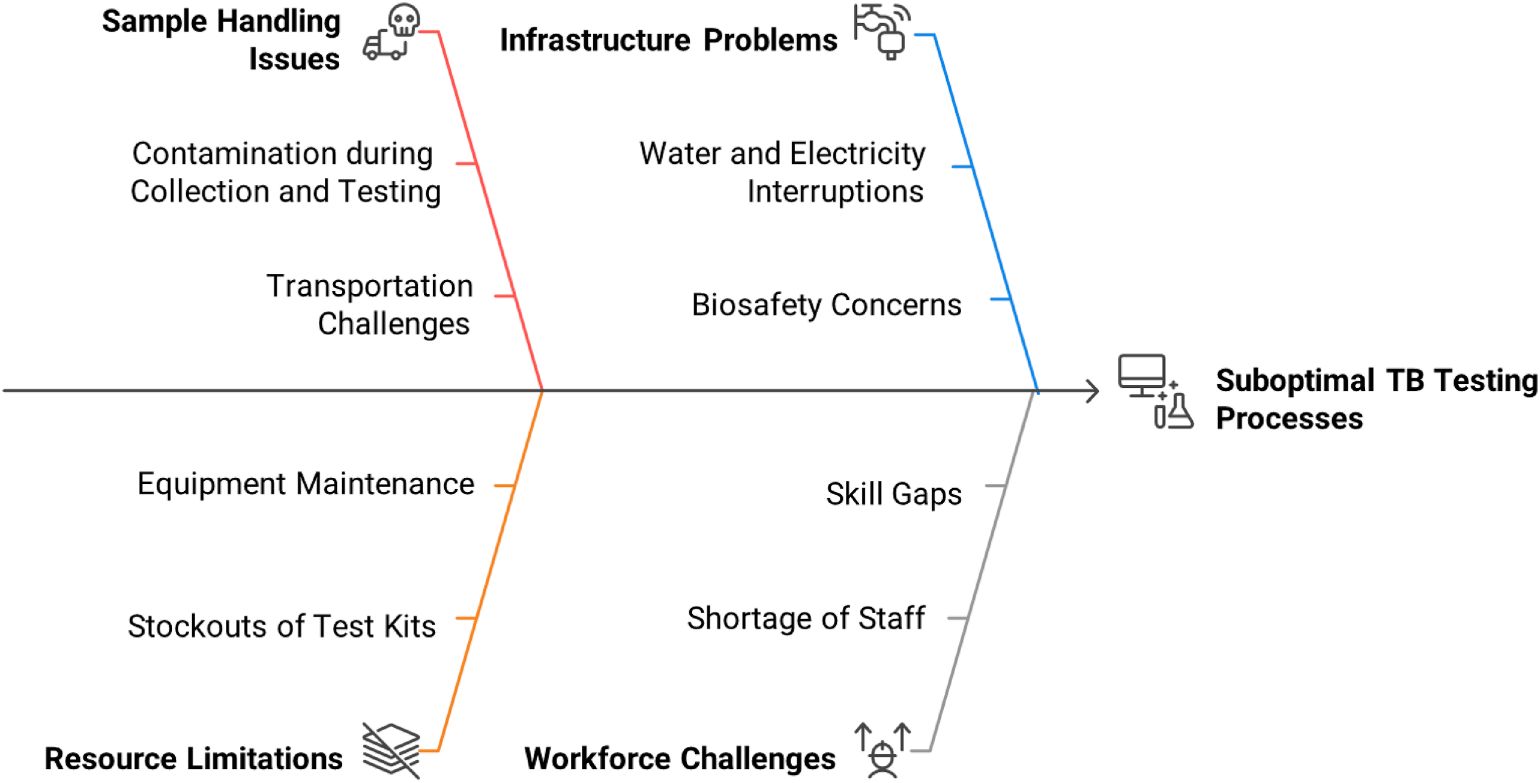
Challenges in TB laboratory testing processes.

### Systemic issues

Several diagnostic laboratory issues are largely related to system issues with the laboratory network. Only a limited number of laboratories can perform drug susceptibility testing, and unstructured transportation does not support a referral of specimens to a higher-level laboratory. A reliance on external funding sources or inconsistent domestic financing mechanisms negatively affects the supply of laboratory equipment and consumables. Infrastructure issues, including interruptions in water and electricity supply and biosafety concerns, were also noted, along with the challenge of maintaining an adequate workforce (see Table). Already short-staffed, diagnostic laboratories are under pressure from competing priorities and multitasking, which was exacerbated during the COVID-19 pandemic. Project-based employment, frequent rotation, unmet needs of training and mentorship for staff, limit long-term engagement in laboratory work. Furthermore, the information system and slow internet connections leads to a preference for paper-based, or in-person result-sharing. These systemic challenges highlight the need for a strategic approach to laboratory strengthening, which is discussed in the next section.

**TABLE.** Key challenges and solutions for strengthening TB laboratory systems.

Key Challenge	Description	Proposed Solutions
Infrastructure	Frequent power/water outages, lack of biosafety level 3 (BSL-3) labs	Investment in modular laboratory units, strengthening electricity/water supply
Workforce shortage	High turnover, lack of trained personnel	Continuous in-service training programs, financial incentives
Supply management issues	Delayed procurement, stockouts of test kits	Implementing real-time inventory tracking systems with early warning feature, streamlining procurement
Digitalization	Slow adoption of electronic data systems	Adopting/expanding digital health infrastructure, training lab staff

## DISCUSSION

The OR provided invaluable insights into the systemic issues affecting diagnostic and treatment services for drug-resistant TB. Ultimately, laboratory issues are health system issues and require a holistic approach to improve diagnostic services.^3^ Although laboratory was under the same authority as on the clinical side, less attention was paid to the laboratory. This has resulted in the absence of a National Laboratory Strategic Plan (NLSP) in some countries, less frequent training and monitoring of diagnostic capacities, and delayed supply procurement. It takes time to identify lab-related issues, to be shared among multiple stakeholders, and to discuss and draw upon action plans to remedy those challenges. To improve these services, it is imperative to ensure that laboratory capacity building receives the same level of attention and resources as clinical management.

The OR was a practical opportunity for participating countries to identify systems issues regarding diagnostic and treatment service delivery. Key challenges identified require coordinated national and international strategies, but these can be addressed by first sharing the identified problems with multiple relevant stakeholders so that there is a common understanding of the problem. Once the political will is gathered, strategic action plans should be developed by the Ministry of Health and National TB Program (NTP)/National TB Reference Laboratory (NTRL) to address the issues. This should be complemented by consideration of how and where to best utilize external support available through various organizations.

Although national governments should take the lead in strengthening TB laboratory systems, targeted international support is crucial in specific areas such as introducing new diagnostic technologies, capacity building, and ensuring quality assurance. Harmonizing efforts among global organizations, supporting capacity building, and improving supply chain management are essential to ensure that NTP/NTRL can effectively diagnose and treat drug-resistant TB reliably and without unnecessary delay. In alignment with the WHO consolidated guidelines on tuberculosis, national TB programs should integrate rapid molecular diagnostics and systematic external quality assurance measures to enhance diagnostic accuracy and efficiency.^2^ By working together, international partners can address systemic challenges and improve the overall effectiveness of global TB control efforts.

## CONCLUSION

The findings from the LIFT-TB project highlight the need for a strategic and coordinated response from national health authorities and international stakeholders supporting the laboratory, clinical, and programmatic sides of implementation. The development and implementation of the NLSP should be prioritized, along with regular training and timely procurement processes to ensure that laboratories can perform essential TB diagnostics. This must be coupled with efforts to address workforce challenges, infrastructure weaknesses, and the slow adoption of digital information systems. International support remains crucial to supplementing national efforts, particularly in global diagnostic expansion.^2^ However, rather than merely emphasizing the international community's role, a more integrated approach should be taken in-country. International organizations, such as WHO, The Global Fund, and others, should coordinate efforts to help countries align national strategies with WHO guidelines and ensure sustainable lab services. This collaborative effort will be key to overcoming the systemic challenges identified and achieving long-term improvements in global TB control.

In conclusion, addressing systemic TB laboratory challenges requires integrating capacity building into national strategies with sustained political commitment and international collaboration, ensuring effective DR-TB diagnosis and treatment. In addition, international organizations (such as WHO and The Global Fund) should help countries to align their national strategies with WHO guidelines. Only by addressing these systemic challenges can we hope to improve the diagnosis and treatment of drug-resistant TB and ultimately reduce the global burden of this deadly disease.
